# Denaturation Behavior and Kinetics of Single- and Multi-Component Protein Systems at Extrusion-Like Conditions

**DOI:** 10.3390/polym12092145

**Published:** 2020-09-20

**Authors:** Maria Quevedo, Heike P. Karbstein, M. Azad Emin

**Affiliations:** Institute of Process Engineering in Life Sciences, Chair of Food Process Engineering, Karlsruhe Institute of Technology, 76131 Karlsruhe, Germany; maria.quevedo@kit.edu (M.Q.); heike.karbstein@kit.edu (H.P.K.)

**Keywords:** whey proteins, α-Lactalbumin, β-Lactoglobulin, whey protein isolate (WPI), denaturation, reactivity, thermomechanical treatment, kinetics, extrusion processing, phase separation

## Abstract

In this study, the influence of defined extrusion-like treatment conditions on the denaturation behavior and kinetics of single- and multi-component protein model systems at a protein concentration of 70% (*w/w*) was investigated. α-Lactalbumin (αLA) and β-Lactoglobulin (βLG), and whey protein isolate (WPI) were selected as single- and multi-component protein model systems, respectively. To apply defined extrusion-like conditions, treatment temperatures in the range of 60 and 100 °C, shear rates from 0.06 to 50 s⁻^1^, and treatment times up to 90 s were chosen. While an aggregation onset temperature was determined at approximately 73 °C for WPI systems at a shear rate of 0.06 s⁻^1^, two significantly different onset temperatures were determined when the shear rate was increased to 25 and 50 s⁻^1^. These two different onset temperatures could be related to the main fractions present in whey protein (βLG and αLA), suggesting shear-induced phase separation. Application of additional mechanical treatment resulted in an increase in reaction rates for all the investigated systems. Denaturation was found to follow 2.262 and 1.865 order kinetics for αLA and WPI, respectively. The reaction order of WPI might have resulted from a combination of a lower reaction order in the unsheared system (i.e., fractional first order) and higher reaction order for sheared systems, probably due to phase separation, leading to isolated behavior of each fraction at the local level (i.e., fractional second order).

## 1. Introduction

Due to their nutritional and functional properties, proteins are often used to produce protein-based products such as sports beverages, meat substitutes, baked products and dairy infant formulae. From a nutritional point of view, protein consumption is necessary for normal growth and repair of body cells, functioning of muscles, and health related immune functions [1]. Furthermore, the unique functionality of proteins includes the ability to form gels, increase the viscosity of solutions, and stabilize emulsions and foams. The functional properties of proteins strongly depend on the molecular structure, and by this, on their physicochemical properties [2]. Thermal, chemical, or mechanical treatment may lead to changes in the molecular structure of proteins, and consequently on their functionality. Therefore, processes such as extrusion have been used to produce protein-based emulsifiers and thickeners [3,4]. Extrusion processing uses shear, heat, and pressure to change the structure of food components, including proteins [5]. Different unit operations such as transport, mixing, shearing, and heating are possible within the extruder, making it a continuous flow reactor. This leads to a reduction of processing steps and acceleration of chemical reactions improving the productivity and energy consumption [6]. Heat transfer from the heated barrel, conversion of mechanical energy into heat energy, and shearing from the rotation of the screws result in thermal and mechanical stresses acting simultaneously on the highly concentrated protein matrices (concentration > 30%). Thus, factors including screw and die configuration, screw speed, flow rate, and barrel heating influence the reactions taking place, i.e., protein denaturation. The native structure unfold, reactive sites hidden in the globular structure become available and new protein–protein interaction are formed, which can lead to protein aggregation. Although there is plenty of information about the effect of temperature and shear rate on the denaturation of proteins, most studies are mainly focused on protein solutions (concentration below 10%), which are quite different from the conditions during extrusion processing.

Since a better understanding of the denaturation behavior of proteins subjected to various process conditions can be used to develop process-engineering approaches to design whey proteins with new functionalities, information about the effect of combined thermal and mechanical treatment on the denaturation reaction and kinetics of proteins at protein concentrations above 30% is needed. Accordingly, in our recent study [7] the influence of protein concentration, treatment temperature, and shear rate on denaturation and aggregation kinetics of β-Lactoglobulin (βLG) as a model protein system at concentrations above 50% (*w/w*) was investigated. The results showed that denaturation and aggregation reaction rates decreased with increasing protein concentration. In comparison to thermal treatment alone, a combination with mechanical treatment resulted in higher reaction rates and consequently, in higher degrees of non-disulfide covalent bond aggregation. Denaturation of βLG at high concentration was found to follow 2.15-order kinetics, which was significantly higher than the reaction order of 1.5 determined for βLG solutions at concentrations below 10%. These results showed that the denaturation and aggregation kinetics for highly concentrated systems differed from the known kinetics for diluted systems. Moreover, in comparison to βLG, whey proteins are expected to show different reaction behavior as it is a multi-component system including various protein fractions (i.e., βLG and α-Lactalbumin (αLA)) as well as non-protein components such as lactose. Therefore, the objective of this study was to systematically investigate the effect of extrusion-like treatment conditions on the denaturation kinetics of a highly concentrated multi-component protein system such as whey protein isolate. To gain better insights about the denaturation mechanisms at high protein concentrations in some way representative for low moisture extrusion for the creation of structures from protein powders, the role of the main whey protein fractions (i.e., βLG and αLA) were also separately investigated. For this, the influence of the shear rate on the reaction onset temperature for βLG, αLA, and whey protein isolate at a concentration of 70% was investigated. Furthermore, the degree of denaturation for the chosen whey protein systems was analyzed and described by a mathematical model to determine the kinetic parameters as a function of temperature, shear rate, and time.

## 2. Materials and Methods

### 2.1. Materials

Whey protein isolate (WPI), German Prot 9000, with a content of 90% protein on dry matter was kindly provided by Sachsenmilch Leppersdorf GmbH (Sachsenmilch Leppersdorf, Leppersdorf, Germany). According to the supplier, the contents of the minor ingredients were ≤ 3% lactose and ≤ 1% fat. Total ash content was 4% and the content of moisture was ≤ 5%. Pure bovine βLG was produced at the Chair of Food and Bioprocess Engineering of the Technical University Munich at Freising-Weihenstephan, Germany. The βLG used in this study was highly pure (< 0.05% lactose, minerals < 0.7%), the moisture content was < 4%, the degree of nativity was > 90%, with 99% protein on dry matter, and the content of βLG in the dry matter was > 98%. Agropur Inc. (Agropur, WI, USA), kindly provided α-Lactalbumin (αLA), BiPro Alpha 9000. According to the supplier, the αLA used in this study was highly native and pure (< 0.2% lactose), the moisture content was < 5.6%, 97.6% protein on dry matter, and the content of αLA in the dry matter was 92.2%.

### 2.2. Sample Preparation

WPI, βLG, and αLA were mixed with deionized water (Millipore Sigma, Burlington, MA, USA) to produce protein doughs with a protein concentration of 70% (*w/w*) with a Thermomix (Vorwerk, Wuppertal, Germany) for 3 min. To ensure a homogenous distribution of water, the samples were stored at 8 °C at least for two days before any experiment.

### 2.3. Defined Extrusion-Like Treatment Conditions and Inline Rheological Analyses

Defined treatments at extrusion-like conditions and inline rheological measurements were performed by a closed cavity rheometer (CCR) (RPA flex, TA Instruments, New Castle, DE, USA) as described in our previous study in detail [7].

To determine reaction onset temperatures, temperature sweep analyses within a temperature range of 30–180 °C were carried out. The measurements were performed at a heating rate of 5 K min⁻^1^ with a shear rate of 0.06 s⁻^1^. The effect of mechanical treatment at a shear rate of 0.06 s⁻^1^ can be neglected, as this shear rate corresponds to the (linear-viscoelastic) LVE region of the samples. To investigate the influence of shear rate on the reaction onset temperature, temperature sweep analysis were also performed in the same temperature range of 30–180 °C and heating rate of 5 K min⁻^1^ at higher shear rates of 25 and 50 s⁻^1^. Isothermal time sweep measurements were conducted to investigate the influence of time, temperature, and shear rate on the reactions taking place, and correspond to defined extrusion-like treatment conditions (i.e., thermal and mechanical treatment) depending on the chosen parameter settings. Samples were treated at temperatures between 60 and 100 °C, shear rates 0.06, 25, and 50 s⁻^1^, and treatment times up to 90 s, respectively. It should be noted that due to the small volume of the measuring chamber (4.5 cm^3^) and preheated lower and upper geometries, the samples achieved the chosen treatment temperatures almost instantaneously. Although during the thermomechanical treatments, the temperature of the chamber geometry is controlled by electric heating and forced air-cooling, at very high protein concentrations and shear rates, viscous dissipation energy led to slightly higher chamber temperatures than the temperatures set. Therefore, the treatment temperatures in the results section are given as a mean value with standard deviation to include this effect. After defined extrusion-like treatment in the CCR, the samples were dried in a vacuum dryer (Heraeus, Hanau, Germany) at 25 °C and 10 mbar and milled with a coffee mini grinder (KYG Group, Dongguan, China) to a particle size <500 μm. For further offline analyses, only the dried protein powders were used. All measurements were conducted at least in duplicate.

### 2.4. Degree of Denaturation

The amount of native protein remaining in solution after the treatment was measured using UV–Vis spectroscopy to determine the degree of denaturation. To induce precipitation of denatured proteins, samples (1 mg mL⁻^1^) were dissolved in an acetate buffer (0.5 M, pH 4.6). After an extraction time of one hour, the denatured proteins were removed by centrifugation at 4301× *g* for 60 min; ensuring that only soluble, and by this, native fractions remained in the supernatant. Absorption of the samples was measured at a wavelength of 280 nm by an Evolution 201 spectrophotometer (Thermo Fischer Scientific Inc., Waltham, MA, USA). Untreated samples were used as reference and every extraction was performed in duplicate for each sample. Using a calibration curve, the concentration in the supernatant was calculated from the measured absorption. The degree of denaturation (*D_D_*) (Equation (1)) was calculated as the protein concentration ratio after (*C_T_*) thermomechanical treatment and before treatment (*C*_0_).

(1) $$D_{D}=\left(\left(\frac{C_{0}-\;C_{T}}{C_{0}}\right)*100\right)$$

### 2.5. Kinetic Modeling

The effect of the heat and shear treatment on the denaturation kinetics was described using formal reaction kinetics. The kinetic parameters were determined using a non-linear regression (NLR) method as described by [7]. An integrated form of the general reaction rate (Equation (2)) can describe the rate of protein denaturation:

(2) $$\left(\frac{C_{T}}{C_{0}}\right)={\left(1+\left(n-1\right)k_{n}C_{0}{}^{n-1}t\right)}^{\frac{1}{1-n}}$$

where *C*_0_ and *C_T_* are the concentrations (g g^−1^) of native whey proteins before and after treatment, respectively, *n* is the overall order of the reaction and *k* is the apparent reaction rate constant ((g g^−1^)^(1−*n*)^ s^−1^). To determine the kinetic parameters with respect to time, data ($\frac{C_{T}}{C_{0}}$ versus *t*) was fitted directly into Equation (2) and *n* and *k* were determined simultaneously by NLR (OriginPro 2018, OriginLab Corporation, Northampton, MA, USA). The overall fit of the model to the experimental data was given by R^2^.

Using the Arrhenius Equation (Equation (3)), the temperature dependence of the reaction rate can be described:

(3) $$k_{T,n}=k_{o,T,n}e^{\left(-\frac{E_{T,A}}{RT}\right)}$$

where *T* is the treatment temperature (K), *R* is the universal gas constant (8.314 kJ kmol K^−1^), *k_T,n_* is thermal-induced rate constant, *k*_0,*T,n*_ pre-exponential factor, and *E_T,A_* is the activation energy (kJ mol^−1^). Since additional shear stress is also expected to influence the reaction kinetics of highly concentrated whey protein systems [7], the modified Arrhenius equation developed from a Boltzmann distribution of mechanical energy in a shear field [8] (Equation (4)) was used to describe the influence of shear stress on the denaturation reaction kinetics:

(4) $$k_{\tau ,n}=k_{o,\tau ,n}e^{\left(-\frac{E_{\tau }}{aJ}\right)}$$

where *k_τ,n_* is the reaction rate constant for the shear-induced reaction and *k_τ_*_,0_ is a constant analogous to *k*_0,*T,n*_ in the Arrhenius equation. *E_τ_* is shear activation energy, *J* is the average rate of shear energy input, and the factor a relates *J* to the average amount of mechanical energy temporarily stored in a mole of chain bonds of the sheared system. Assuming that the shearing reduces the thermal activation energy necessary for protein unfolding, then, the resulting rate constant can be formulated using a modified Arrhenius equation (Equation (5)):

(5) $$k=k_{o}e^{\left(-\frac{E_{t}}{RT}\right)}=k_{o}e^{\left(-\frac{E_{0}\;-\;\Delta E}{RT}\right)}$$

With

(6) $$\Delta E=\;E_{0}-\;E_{t}$$

where *E_t_* is the total activation energy, *E*_0_ is the thermal activation energy in the absence of shear stress and Δ*E* activation energy reduction resulting from the mechanical treatment. Using Equation (6) and the reaction constant calculated from Equation (3), the total activation energy was calculated. *E*_0_ was calculated using a shear rate of 0.06 s^−1^, as at this shear rate the influence of mechanical treatment on the reactions is negligible. Then activation energy reduction was then calculated using Equation (6).

## 3. Results and Discussion

### 3.1. Influence of Defined Thermal and Mechanical Treatment on the Reaction Behavior and Onset Temperatures of Whey Proteins

**Figure 1:**
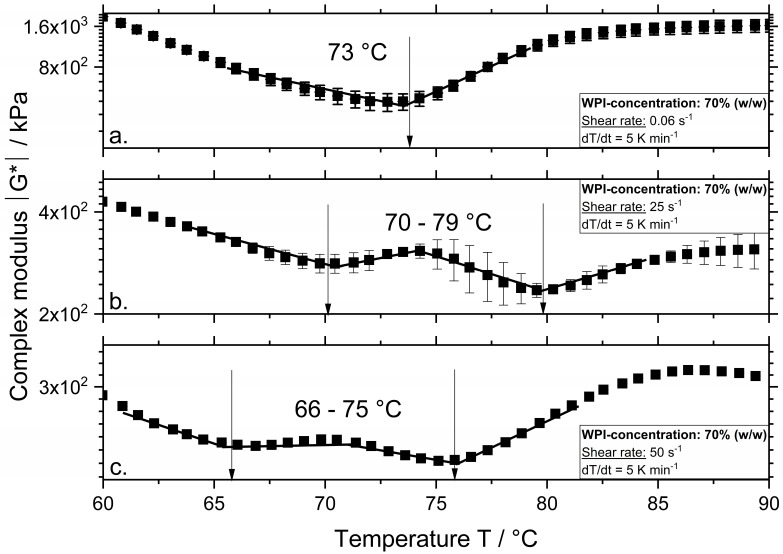
Complex modulus $\left|G^{*}\right|$ as a function of temperature for systems with a whey protein isolate (WPI) concentration of 70% (*w/w*) and a shear rate of 0.06 (**a**), 25 (**b**), and 50 s^−1^ (**c**). Measurements were performed at a constant heating rate of 5 K min^−1^.

The complex modulus $\left|G^{*}\right|$ is depicted in Figure 1 as a function of temperature and shear rate for the system containing 70% (*w/w*) WPI. The complex modulus in Figure 1a shows two distinct regions: initial decrease (Region I) followed by an increase (Region II). The decrease in the complex modulus in Region I was related to the increased mobility of the protein molecules due to increasing temperature. A distinct change in the slope happened at approximately 73 °C (transition from Region I and II), which is defined as the aggregation onset temperature [9,10]. Although the aggregation might have started at lower temperatures, this is the temperature where the aggregation outweighs the effect seen in Region I. By increasing the shear rate to 25 and 50 s^−1^, the course of the complex modulus changed compared to Figure 1a. The complex modulus in Figure 1b,c shows four distinct regions: initial decrease (Region I), followed by an increase (Region II), a further decrease (Region III), and final increase (Region IV), which led to the determination of two aggregation onset temperatures.

At a shear rate of 25 s^−1^, the aggregation onsets at 70 and 79 °C. By increasing the shear rate to 50 s^−1^, these temperatures decreased to 66 and 75 °C. The occurrence of two onset temperatures when the samples were sheared might suggest shear induced phase separation of proteins. At a shear rate of 0.06 s^−1^, the protein molecules of different fractions (i.e., βLG and αLA) were homogenously distributed, and the measured onset temperature implied that they involve in the aggregation reaction simultaneously. At higher shear rates, it is possible that these protein fractions form separated phases due to their limited miscibility, which might lead to variations of the molecular mobility, interactions, and aggregation at the local level. This possibility is discussed in the following sections in more detail.

**Figure 2:**
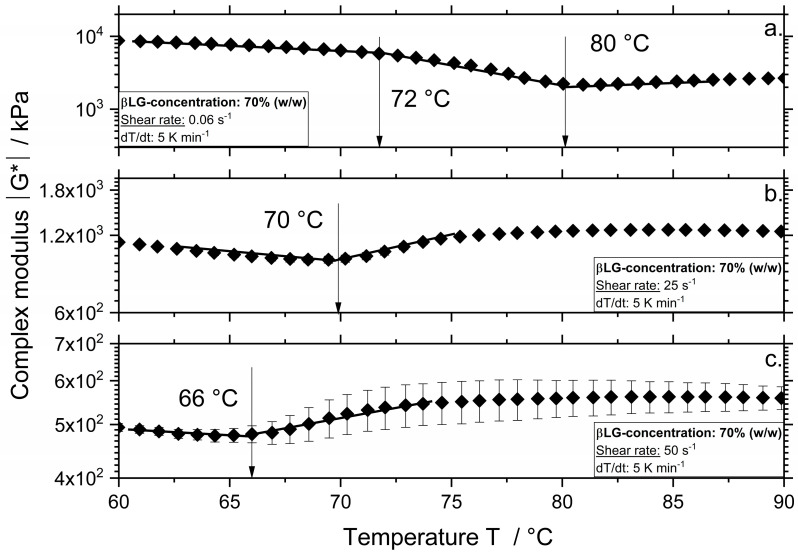
Complex modulus $\left|G^{*}\right|$ as a function of the temperature for systems with a βLG concentration of 70% (*w/w*) and a shear rate of 0.06 (**a**), 25 (**b**), and 50 s^−1^ (**c**). Measurements were performed at a constant heating rate of 5 K min^−1^.

**Figure 3:**
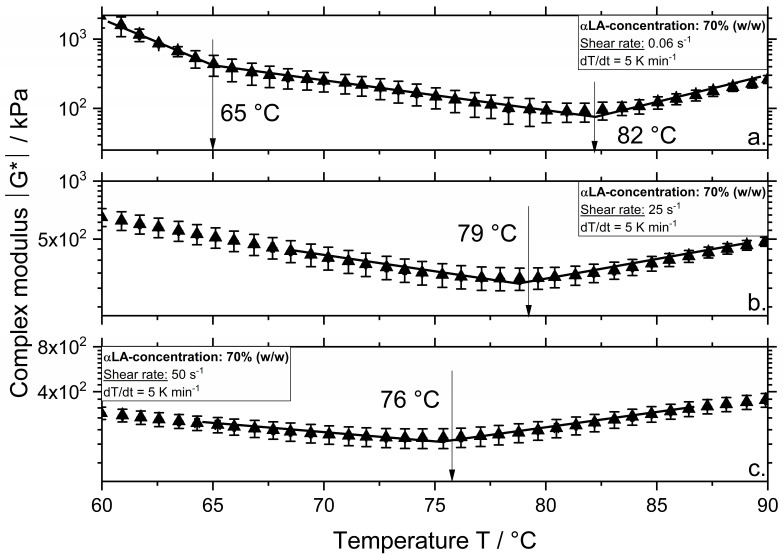
Complex modulus $\left|G^{*}\right|$ as a function of temperature for systems with a αLA concentration of 70% (*w/w*) and a shear rate of 0.06 (**a**), 25 (**b**), and 50 s^−1^ (**c**). Measurements were performed at a constant heating rate of 5 K min^−1^.

The results for βLG and αLA samples depicted in Figure 2a and Figure 3a show that there were two temperatures at which the course of the complex modulus changed. These temperatures are defined as the denaturation and aggregation onset temperature, respectively. As shown in Figure 2a, the denaturation onset temperature for βLG with a concentration of 70% was approximately 72 °C, which is in accordance with previous results in [9]. In the case of αLA (Figure 3a), a lower denaturation of approximately 65 °C was measured. For both proteins, increasing the shear rate (depicted in b, c) led to a decrease in the aggregation onset temperature.

In contrast to WPI, which is a complex system containing more than one protein fraction, the aggregation onset temperature at a shear rate of 0.06 s^−1^ for systems containing the single fractions αLA and βLG increased from 73 to 82 °C and 80 °C, respectively. As expected, αLA shows higher aggregation onset temperatures compared to βLG due to its four stabilizing disulfide bonds and lacking a free thiol group. Higher aggregation temperatures were observed for αLA compared to βLG from various authors at various treatment conditions [11,12]. The results of WPI at 0.06 s^−1^ show that the βLG had a lower aggregation temperature in this complex system. Since the WPI systems show lower viscosity as the βLG systems, higher reaction rates were expected due to an increased molecular motion, which explained the lower onset temperature. Additionally, an increase in reaction rate was expected as in WPI systems all protein fractions present could participate and form aggregates. Although no denaturation onset temperature was observed for the WPI at a shear rate of 0.06 s^−1^, it was expected that denaturation onsets between 60 and 70 °C.

In accordance to these results, [13] showed that the denaturation onset temperature of βLG decreased when βLG was combined with αLA. The authors also showed that systems containing only αLA showed a denaturation temperature of approximately 65 °C. Since αLA denatured at lower temperatures compared to βLG, it seems possible that in homogenous systems containing both fractions, the aggregation reactions starts from a reactive αLA monomer if the thermal treatment is high enough to activate the αLA monomers. Furthermore, the concentration of each fraction present in the mixture was also expected to influence denaturation and aggregation reactions.

At a shear rate of 25 s^−^^1^, βLG shows an aggregation onset temperature of 70 °C and αLA of 79 °C. Increasing the shear rate to 50 s^−^^1^ led to a decrease in the aggregation onset temperature to 66 and 76 °C, for βLG and αLA, respectively. This effect was also observed in our previous studies on WPI and βLG [7,10]. It seems that the aggregation onset temperatures determined for the sheared WPI systems corresponded to the onset temperatures of the single protein fractions. This can be related to the phase separation phenomena, which has been observed for various biopolymer mixtures [14]. For instance, [15] reported for βLG and κ-carrageenan (κ-Car) systems two slopes of the elastic modulus G’ during a heating/shearing and cooling down treatment step. The first slope corresponded to the setting of the βLG network, and the second to the further protein network affected by the formation of the κ-Car gel in the system [16]. Although the authors investigated a mixture of globular proteins and polysaccharide (i.e., βLG and κ-car), similar to the results observed in Figure 1 during heating and shearing of WPI systems, two markedly different rheological behaviors (i.e., a bicontinuous profile) were observed suggesting the formation of phase-separated gels. Similarly, the first onset temperature suggests the setting of βLG aggregates and the second one, the aggregation of αLA affecting the previously formed βLG network.

Although phase separation of protein solutions has not been often reported, it is indeed expected to occur with time [17]. Since proteins have, like all polymers or all solid materials, a limited solubility, at a protein concentration above their solubility, separation into different phases occur [18].

### 3.2. Influence of Defined Thermal and Mechanical Treatment on the Denaturation of Whey Proteins

**Figure 4:**
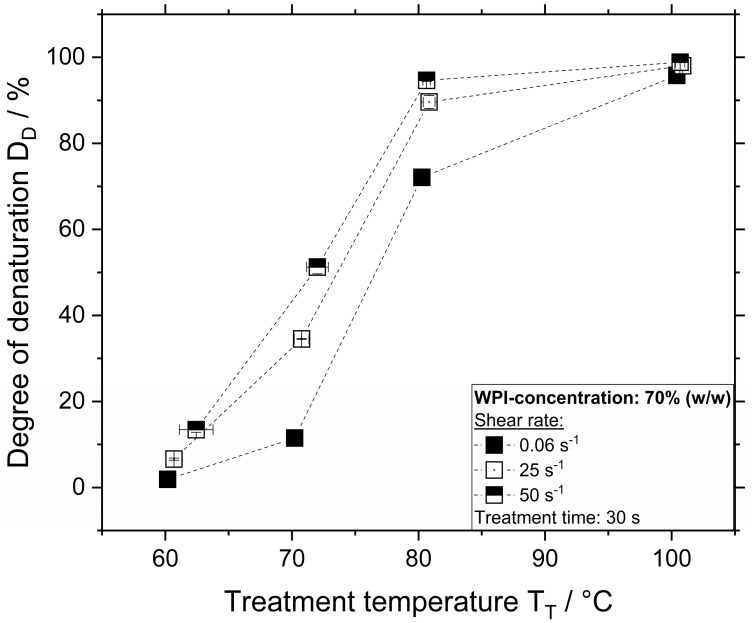
Degree of denaturation as a function of temperature and shear rate for a WPI concentration of 70% (*w/w*) and treatment time of 30 s.

**Figure 5:**
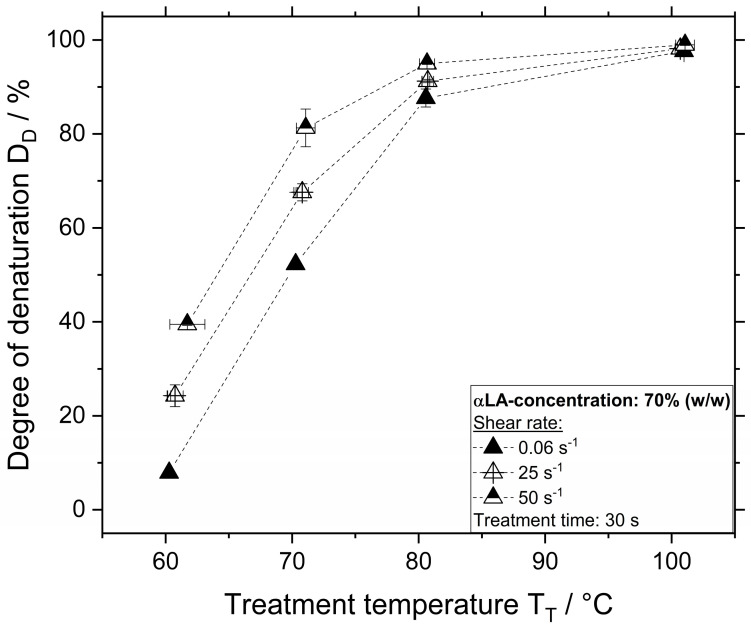
Degree of denaturation as a function of temperature and shear rate for a αLA concentration of 70% (*w/w*) and treatment time of 30 s.

The influence of treatment temperature and shear rate on the *D_D_* for samples containing WPI and αLA at a concentration of 70% (*w/w*) treated for 30 s is depicted in Figure 4 and Figure 5, respectively.

As shown in Figure 4, thermal treatment of WPI at 60 and 70 °C resulted in a degree of denaturation of 2% and 12%, respectively. The degree of denaturation for samples treated thermally at 80 °C was approximately 80%. At higher temperatures, the degree of denaturation was already above 90% even at the lower shear rate of 0.06 s^−^^1^. Increasing the shear rate to 50 s^−^^1^ led to an increase in denaturation for all temperatures investigated. Thermomechanical treatment of WPI at a shear rate of 50 s^−^^1^ and temperatures 60, 70, and 80 °C resulted in a degree of denaturation of approximately 13%, 51%, and 94%, respectively. At temperatures above 100 °C, the effect of the shear rate on the denaturation was no longer visible as at these conditions the denaturation reaction was already high.

As shown in [7], the thermal treatment of βLG at a concentration of 70% resulted in similar denaturation compared to WPI. Treatment of βLG systems at 60, 70, and 80 °C resulted in a degree of denaturation of approximately 2%, 11%, and 72%, respectively, whereas thermomechanical treatment at a shear rate of 50 s^−^^1^ and temperatures 60, 70, and 80 °C resulted in a degree of denaturation of approximately 22%, 58%, and 96%, respectively.

Although the denaturation of βLG and WPI systems at a shear rate of 0.06 s^−^^1^ was very similar, at higher shear rates the βLG systems showed higher degrees of denaturation. Probably due to the fact that the βLG systems show higher values of complex modulus and by this, of viscosity (Figure 1 and Figure 2), therefore the effect of the shear stresses on the reactions is expected to increase, which could then lead to higher reaction rates.

As depicted in Figure 5, the degree of denaturation of αLA was higher compared to WPI and βLG independent of the applied treatment temperature or shear rate. At a shear rate of 0.06 s^−^^1^, treatment at temperatures of 60, 70, and 80 °C resulted in approximately 7%, 52%, and 87%, respectively. At a shear rate of 50 s^−^^1^, even a treatment at 60 °C resulted in approximately 40% denaturation. Samples treated at 70 °C and 50 s^−^^1^ were over 80% denatured. Higher treatment temperatures resulted in almost a complete denaturation since the degree of denaturation was above 80% even at the lowest shear rate of 0.06 s^−^^1^.

Although the denaturation temperature for aLA solutions determined using differential scanning calorimetry (DSC) was approximately 65 °C [11,13,19,20,21], when αLA was heated at defined temperatures (below 100 °C), reversible denaturation through the formation of non-covalent bonds was observed. Due to the weak nature of these bonds, the newly formed bonds break up as soon as samples cool down, αLA returns to a quasi-native state, and renaturation takes place [22].

The overall high values of denaturation observed in Figure 5 could result either from the high concentration of αLA or from the small amount of βLG (approximately 5%) present in the αLA protein powder. If the reaction rate is high enough, which is the case at high protein concentrations, then the intermolecular thiol-disulfide exchange is rapid and the probability of renaturation decreases [11], leading to high values of denaturation. Additionally it is possible, that the small amount of βLG present in the αLA protein powder could act as a catalyst for the reactions, making a protein molecule available with a free thiol group, which then could react with an αLA molecule, initiating the aggregation reaction.

### 3.3. Kinetics of Denaturation of Whey Proteins at Extrusion-Like Conditions

Data from the results of the degree of denaturation was plotted and fitted using NLR to obtain the kinetic parameters shown in Table 1 and Table 2.

The kinetic parameters for the denaturation reaction for αLA after thermal and mechanical treatment at a concentration of 70% (*w/w*) are shown in Table 1. For the investigated range of treatment conditions, a denaturation reaction order of 2.262 was identified. The reaction rate constant (*k*) increased with temperature and a combination of thermal and mechanical treatment led to even higher values of *k*. The reaction rate determined in [7] for βLG at concentrations between 50% and 70% was 2.151, which is very similar to the reaction rate shown in Table 1.

In contrast, the reaction order for WPI at a concentration of 60% was 1.909 [10]. A similar reaction order (1.865) was determined when the concentration of the WPI system increased to 70%. Although compared to our previous results the denaturation reaction order decreased slightly from 1.909 to 1.865 when the concentration of WPI increased from 60% to 70%, the accelerating effect of temperature and shear rate on the reactions was still observed. There is clearly a dependence of protein fractions present in the protein system on the reaction order. The reaction order for single protein fractions follows a fractional second order, whereas for complex systems such as WPI where multiple protein fractions are present, a fractional first order was observed. In theory, a second order is observed when the reactions depend on the concentration of two reactants or one concentration squared. For βLG it was hypothesized that a reactive monomer is needed to start the reaction, consequently starting from dimers, then native monomers are formed, which then are activated and react further with other monomers to form non-native dimers, trimers, and eventually oligomers [23]. Consequently, the denaturation reaction depends on the concentrations of dimers and reactive monomers available. Denaturation of αLA is expected to follow a similar mechanism, starting from monomers, which need a monomer with a free thiol group to react. When the thermal treatment is high enough the disulfide bonds stabilizing the αLA molecule are broken down. As shown in [12], thermal treatment at 100 °C for 10–30 min results in up to a 25% loss of disulfide bonds, which could lead to the formation of reactive groups similar to thiols. Similar to βLG, the reactive αLA monomers react then with monomers and form disulfide bonded oligomers through thiol-disulfide exchange reactions. For the WPI systems, either the free thiol group of the βLG can react with βLG monomers or αLA monomers, therefore the reactions depend mostly on the concentration of the thiol groups available resulting in a fractional first order. Although for WPI a first fractional order reaction was observed, the reaction order is higher than most of the findings of previous studies for lower protein concentrations at neutral pH. Most of the kinetic data for the denaturation reaction of whey proteins takes only into consideration the thermal denaturation of βLG in the system and a reaction order of 1.5 is frequently observed. The increase in the reaction order at the investigated conditions could arise either from the increased protein concentration or from the additional effect of shear stresses on the reactions. As shown in Figure 1, for systems containing multiple protein fractions, a combination of thermal and mechanical treatment led to a more complex reaction behavior as two aggregation onset temperatures were observed. The reaction order of almost 2 (1.865 and 1.909) could then result from a combination of a lower reaction order for unsheared systems and higher reaction order for sheared systems as phase separation occurs, leading to similar reaction orders as for the single protein fractions. Consequently, it is expected that the denaturation reaction rate after thermomechanical treatment of complex systems containing various protein fractions depends on the ratio and concentration of each fraction. As already shown for less concentrated systems, changes of βLG and αLA ratios in WPI systems result in aggregates with different protein composition [24].

The calculated values of the activation energy (*E_t_*) for αLA and WPI systems at a concentration of 70% are depicted in Table 2. The results show that the activation energy decreased with increasing thermal and mechanical treatment. Similarly results are shown in [7] for βLG systems with a concentration of 70%. As expected from the previously discussed results, in the temperature range of 80–100 °C the activation energy was highest for βLG (107–133 kJ mol^−1^) [7], then for αLA (109–117 kJ mol^−1^) followed by WPI (80–100 kJ mol^−1^). Since at temperatures above 80 °C both of the protein fractions (αLA and βLG) are expected to aggregate, the energy input in the system due to the treatment was high enough to induce aggregation, consequently the activation energy was lowest for WPI systems. In the temperature range between 60 and 80 °C, the activation energy was lowest for the αLA systems. Since αLA denatured at approximately 65 °C, treatment in this temperature range positively affected the reaction leading to lower values of activation energies. Similar results were observed in [25] since the denaturation of αLA in solutions was found to be initially even faster than that of βLG at 65 °C. The maximum reduction of the activation energy at a shear rate of 50 s^−1^ was 19% and 12% for αLA and WPI, respectively. In WPI systems, the intermolecular interactions between αLA and βLG possibly resulting in phase separation, led to higher values of activation energy and the effect on the shear rate on the reduction of activation energy seemed to decrease. Furthermore, for αLA systems by increasing the shear rate from 25 to 50 s^−1^, the activation energy decreased by only 1%, which could imply that shear stress indeed influences the reactions, but only up to a limit and the maximum reduction is achieved at the chosen shear rates. The activation energy determined for the βLG systems was overall higher than for αLA and WPI. As already shown in the results from temperature sweeps, the complex modulus of the βLG systems was higher than for αLA and WPI systems. The high complex modulus and by this high viscosity led to low molecular mobility, lower reaction rates, and higher values of activation energy. Therefore, the calculated activation energies for WPI systems and the effect of the shear rate on its reduction represent the combined effect observed for the main fractions, βLG and αLA. Similar results were observed by [26], since the denaturation rate for βLG was lower than for systems containing more than one whey protein fraction. Overall, the determined activation energies are in agreement with previously reported values by other authors [26,27]. In the temperature range below and above 80 °C, the activation energy values for various whey protein systems ranged from 189 to 283 kJ mol^−1^ and between 80 and 133 kJ mol^−1^, respectively.

## 4. Conclusions

Using the closed cavity rheometer investigation on the influence of defined extrusion-like treatment conditions on the denaturation behavior and kinetics of single- and multi-component protein model systems at protein concentration of 70% (*w/w*) was possible. Results of the temperature sweep analysis showed that for αLA and βLG, corresponding to single-component systems, independent of the shear rate applied one onset temperature was determined. Similarly, for the unsheared multi-component (WPI) system, one aggregation onset temperature was determined. In contrast, when the multi-component system was sheared, two onset temperatures corresponding to each of the main whey protein fractions were observed. A combination of thermal and mechanical treatment led to higher degrees of denaturation for all the protein systems investigated. In contrast to many studies, αLA showed a higher degree of denaturation compared to WPI and βLG even without the additional influence of shear stress. It seems at high protein concentrations, the renaturation of αLA after thermal and mechanical treatment might decrease due to high reaction rates. Since the reaction order for αLA and βLG was very similar (2.262 and 2.151, respectively), the reaction pathways of these proteins under thermomechanical treatment are expected to be similar. For WPI systems, a reaction order of 1.865 was determined, which probably results from a combination of a lower reaction order for unsheared systems and higher reaction order for sheared systems due to phase separation, leading to a reaction order similar to the single protein fractions. Although the determined values of activation energy are in accordance with previous studies, it was shown that the shear stress influenced the denaturation reaction of single- and multi-component protein systems differently. Furthermore, it was shown that additional information about the denaturation mechanisms of whey proteins at high protein concentration could be gained by combining protein chemistry and rheological analysis. Since changes in the concentration and ratio of βLG and αLA could lead to changes in the denaturation and aggregation behavior of complex systems such as WPI, further investigations are needed to fully understand the reactions taking place and eventually be able to control them. Additionally, investigations on the influence of protein denaturation on the resulting nutritional properties, e.g., availability of essential amino acids during digestion, are needed to be able to design protein-based novel ingredients with enhanced functionality and biological activity for desired health outcome.

## Figures and Tables

**Table 1:** Denaturation kinetic parameters for αLA at a concentration of 70%.

Temperature/°C	Shear Rate/s^−1^	Reaction Order n/-	k_T,τ/2.262/_(g g^−1)−1.262^ s^−1^	R^2^/-	αLA/%
60	0.06	2.262 ± 0.007	1.947 × 10^−9^	0.993	70
	25		7.420 × 10^−9^	0.999	
	50		1.565 × 10^−8^	0.999	
70	0.06		2.738 × 10^−8^	0.998	
	25		5.569 × 10^−8^	0.999	
	50		1.291 × 10^−7^	0.996	
80	0.06		2.314 × 10^−7^	0.993	
	25		3.686 × 10^−7^	0.992	
	50		7.594 × 10^−7^	0.993	
100	0.06		1.971 × 10^−6^	0.993	
	25		2.872 × 10^−6^	0.994	
	50		5.609 × 10^−6^	0.995	

**Table 2:** Values of activation energy without shear stress, reduction of activation energy due to shear stress, and their ratio from the denaturation kinetic parameters for αLA and WPI samples with a protein concentration of 70% (*w/w*) at different thermomechanical treatment conditions.

Temperature Range/°C	Shear Rate/s^−1^	Reaction Order n/-	Protein Concentration/%	E_t_/kJ mol^−1^	E_0_/kJ mol^−1^	ΔE/kJ mol^−1^	(ΔE/E_0_)/%
80–100	0.06	2.262 ± 0.007	αLA70	117.239	117.239		
	25			112.381		4.859	4%
	50			109.441		7.799	7%
60–80	0.06			233.653	233.653		
	25			190.844		42.808	18%
	50			189.808		43.845	19%
80–100	0.06	1.865 ± 0.055	WPI70	100.361	100.361		
	25			85.434		14.927	15%
	50			80.411		19.950	20%
60–80	0.06			267.176	267.176		
	25			244.372		22.804	9%
	50			234.644		32.532	12%
